# CD133 Is a Useful Surrogate Marker for Predicting Chemosensitivity to Neoadjuvant Chemotherapy in Breast Cancer

**DOI:** 10.1371/journal.pone.0045865

**Published:** 2012-09-25

**Authors:** Naoki Aomatsu, Masakazu Yashiro, Shinichiro Kashiwagi, Tsutomu Takashima, Tetsuro Ishikawa, Masahiko Ohsawa, Kenichi Wakasa, Kosei Hirakawa

**Affiliations:** 1 Department of Surgical Oncology, Osaka City University Graduate School of Medicine, Osaka, Japan; 2 Oncology Institute of Geriatrics and Medical Science, Osaka City University Graduate School of Medicine, Osaka, Japan; 3 Department of Diagnostic Pathology, Osaka City University Graduate School of Medicine, Abeno-ku, Osaka, Japan; Johns Hopkins University, United States of America

## Abstract

**Background:**

Neoadjuvant chemotherapy (NAC) is a standard care regimen for patients with breast cancer. However, the pathologic complete response (pCR) rate remains at 30%. We hypothesized that a cancer stem cell marker may identify NAC-resistant patients, and evaluated CD133 and ALDH1 as a potential surrogate marker for breast cancer. The aim of this study was to find a surrogate maker to predict chemosensitivity of NAC for breast cancer.

**Methodology/Findings:**

A total of 102 patients with breast cancer were treated with NAC consisting of epirubicin followed by paclitaxel. Core needle biopsy (CNB) specimens and resected tumors were obtained from all patients before and after NAC, respectively. Chemosensitivity and prognostic potential of CD133 or ALDH1 expression was evaluated by immunohistochemistry. Clinical CR (cCR) and pCR rates were 18% (18/102) and 29% (30/102), respectively. Forty-seven (46%) patients had CD133-positive tumors before NAC, and CD133 expression was significantly associated with a low pCR rate (p = 0.035) and clinical non-responders. Multivariate analysis revealed that CD133 expression was significantly (p = 0.03) related to pCR. Recurrence was more frequent in patients with CD133-positive tumors (21/47, 45%) than that in patients with CD133-negative tumors (7/55, 13%). The number of patients with CD133-positive tumors (62%) after NAC was higher than that (46%) before NAC. Furthermore, most patients with CD133-positive tumors before NAC maintained the same status after NAC.

**Conclusion/Significance:**

CD133 before NAC might be a useful marker for predicting the effectiveness of NAC and recurrence of breast cancer after NAC.

## Introduction

Neoadjuvant chemotherapy (NAC) increases the resectability of tumors and decreases the risk of postoperative recurrence; thus resulting in superior long-term survival [1], [2]. For this reason, NAC is a standard care regimen for patients with various types of carcinomas, including breast cancer [3]. The optimal regimen for NAC in breast cancer involves a combination of 5-fluorouracil, epirubicin, and cyclophosphamide (FEC), followed by paclitaxel (PTX). [4], [5] The main aim of NAC is to reduce the size of the primary tumor, increase the likelihood of breast conservation [6], and allow evaluation of the therapeutic effects that facilitate establishment of therapeutic strategies based on the evaluation results [7]. Recent studies have demonstrated that pathologic complete response (pCR) in primary breast tumors after NAC correlates with improved disease-free survival (DFS) and overall survival (OS) [5], [8]. NAC for breast cancer has a pCR rate of approximately 30% [6], [9], [10] and a clinical CR (cCR) rate of approximately 60% [10]. In contrast, NAC is ineffective in approximately half of all patients, and many experience toxicity. Therefore, it would be advantageous to identify patients with chemosensitive tumors before initiating NAC, to avoid potential therapy-related complications and inappropriate delay of surgical treatment.

NAC has numerous advantages, including the use of pathological response data as a surrogate marker for long-term clinical outcome [11], [12] and assessment of responsiveness to NAC that allows the evaluation of potential predictive molecular markers for chemosensitivity. Several biological markers, including the estrogen receptor (ER), progesterone receptor (PgR), HER2, Ki-67, p21, p53, Bcl, multi-drug-resistant P-glycoprotein, and topoisomerase 2A, have recently been investigated; however, there is no clear correlation between marker expression and chemosensitivity after sequential taxane- and anthracycline-based chemotherapies [13]–[17], and more useful predictive markers for chemosensitivity need to be clinically identified.

**Figure 1:**
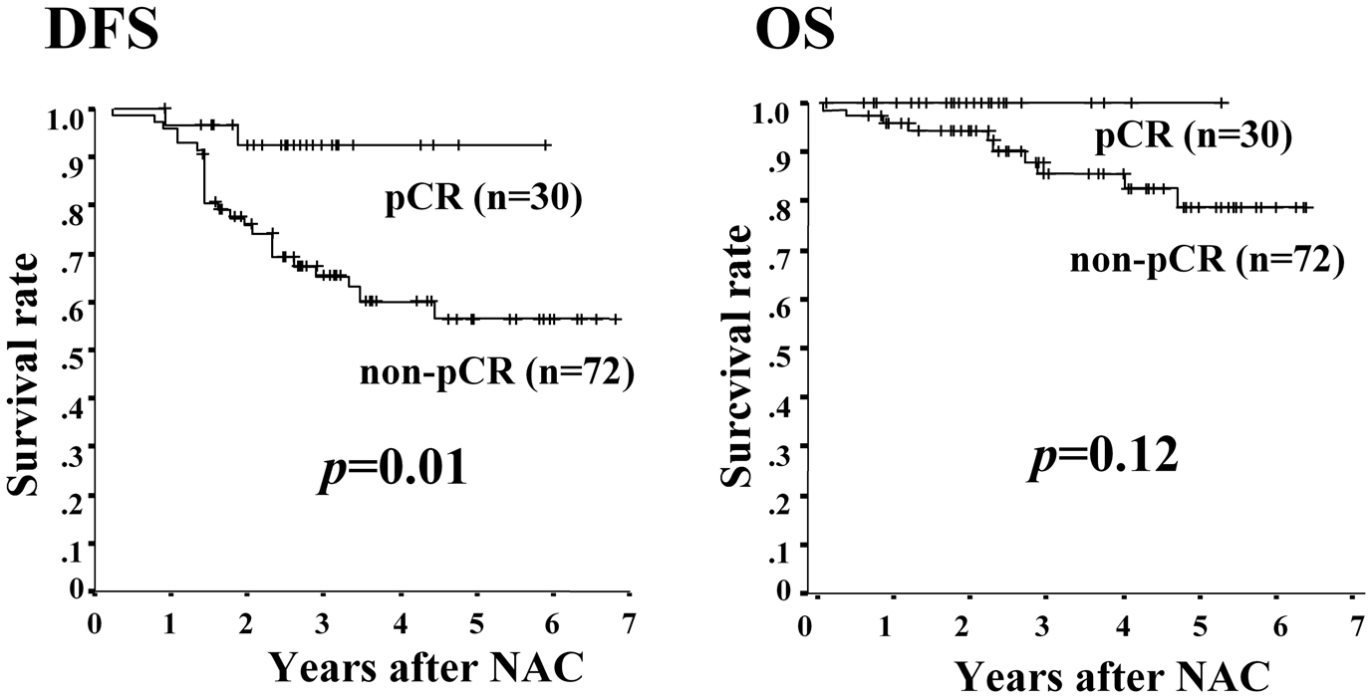
Association between pCR and survival. DFS in pathologic non-responders was significantly (p = 0.01) shorter than that for responders, while OS was not significantly different (p = 0.12).

The recent discovery of the hierarchical organization among cancer stem cells (CSCs) and the finding that cancers emerge from their own progenitor stem cells has had important implications in cancer therapy [18]. In addition to being considered the source of tumor initiation and metastasis [19], [20], CSCs have been demonstrated to be resistant to chemotherapy, indicating that they are also responsible for tumor recurrence [21], [22]. In fact, several in vitro studies have shown that CSCs are resistant to PTX, doxorubicin, 5-fluorouracil, and platinum. Recently, Prominin-1 (CD133) has been considered to be a CSC marker in many types of cancers, such as breast [23]–[25], colorectal [19], [20], brain [26], [27], prostate [28], pancreatic [29], and gastric cancers [30]. In addition, recently, aldehyde dehydrogenase (ALDH) 1 has been identified as a reliable marker for breast CSC marker [31]–[33].

**Table 1:** Clinical and pathological response in 102 primary breast cancers.

clinical response		pathological response	
cCR	17% (18/102)	Grade 1	45% (46/102)
cPR	61% (62/102)	Grade 2	35% (36/102)
cNC	20% (20/102)	Grade 3	20% (20/102)
cPD	2% (2/102)		
		pCR	29% (30/102)

Assesment of clinical and pathological response was described in Materials and Methods.

cCR; clinical complete response, cPR; clinical partial response, cNC; clinical no change, cPD; progressive disease, pCR; pathologic complete response.

In this retrospective study, to evaluate the potential of CD133 or ALDH1 as a surrogate marker for NAC resistance, we examined the correlation between chemosensitivity to NAC and CD133 or ALDH1 as well as prognosis of patients with breast cancer after NAC treatment.

## Patients and Methods

### Patients

A total of 102 patients with breast cancer that was considered to be stage IIA, IIB, and IIIA, was treated with NAC from 2004 to 2009. Tumors were confirmed histopathologically by core needle biopsy (CNB) and were staged by ultrasonography or computed tomography. The clinical stage was based on the TNM Classification of Malignant Tumors, 6th Edition [34]. The tumor size and axillary lymph node metastasis were examined by ultrasonography. No patients had evidence of distant metastasis at the time of surgery. The median age of the patients was 55.0 years old (range 26–78 years old). All of the cases were received neoadjuvant chemotherapy with 4 cycle of 5FU 500 mg/m^2^, epirubicin 75 or 100 mg/m^2^, and cyclophosphamide 500 mg/m^2^ (FEC) followed by 12 cycles of weekly paclitaxel 80 mg/m^2^ (wPTX). Sixteen of 102 patients showed HER2-positive breast cancer, and were administered weekly trastuzumab with wPTX. Patients were underwent mastectomy or breast-conserving surgery after NAC. Patients who underwent breast-conserving surgery were administered postoperative radiotherapy. Overall survival time was set in days as the period from the NAC starting day. This study was conducted with the consent of the ethical committee of Osaka City University, and informed consent was obtained from all subjects.

### Assesment of Clinical and Pathological Response to NAC

Clinical response of primary tumor was assessed by ultrasonography and physical examination after NAC. Clinical responses were classified according to WHO criteria [35]. After NAC, patients underwent appropriate surgery. The clinical response to preoperative chemotherapy was decided from the two diameters measurable in two dimensions determined by multiplying the longest diameter by the greatest perpendicular diameter: clinical complete response (cCR); a disappearance of all known disease determined by two observations not less than four weeks apart, clinical partial response (cPR); a 50% or more decrease in total tumor lesions, clinical no change (cNC); a 50% decrease in total tumor size, nor a 25% increase in the tumor size, clinical progressive disease (cPD); a 25% or more increase in the tumor size, or the appearance of new lesions. cCR and cPR were judged as effective. Pathological response of tumor and dissected lymph nodes were classified according to the evaluation criteria of the Japanese Breast Cancer Society (JBCS) [36], using a 5-grade scale (Grade 0, 1a, 1b, 2, and 3) as follows: Grade 0, no response or almost no change in cancer cells after treatment; Grade 1, slight response; Grade 1a, mild response, mild change in cancer cells regardless of the area, or marked changes in cancer cells in less than one-third of total cancer cells; Grade 1b, moderate response, marked changes in one-third or more but less than two-thirds of tumor cells; Grade 2,marked response or marked changes in two-third or more of tumor cells; and Grade 3,no residual tumor cells, necrosis or disappearance of all tumor cells, or replacement of all cancer cells by granuloma-like and/or fibrous tissue. pCR (pathological complete response) were defined as the complete disappearance of infiltrates, including lymph node infiltrates, with or without intraductal components. Tumors with residual ductal carcinoma in situ were included in the pCR group. Marked changes approaching a complete response with only a few remaining cancer cells were classified as near pCR [37], [38]. The others were grouped in the non-pCR.

**Figure 2:**
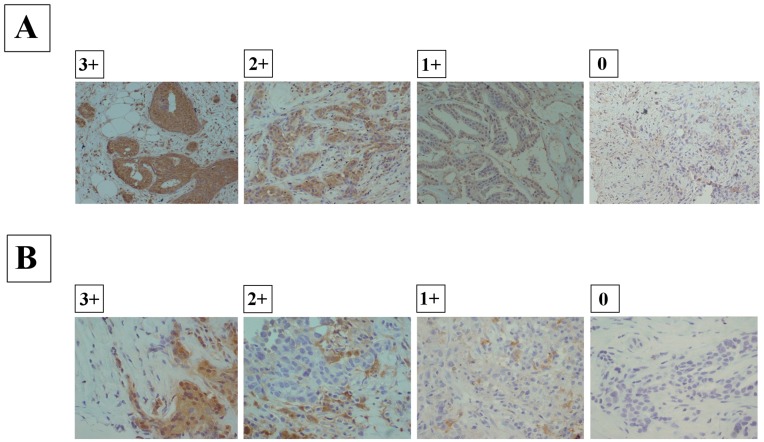
Immunohistochemical staining of CD133 or ALDH1. (A), Immunohistochemical determination of CD133 expression. The CD133 antibody stained intensely at the membrane and in the cytoplasm of cancer cells. Scores were applied as follows: 0, negative staining in all cells; 1+, weakly positive or focally positive staining in <10% of cells; 2+, moderately positive-staining in 10%-50% of cells; and score 3+, strongly positive-staining, involving 50% or more of the cells. (B), Immunohistochemical determination of ALDH1 expression. The ALDH1 antibody stained intensely at the membrane and in the cytoplasm of cancer cells. Scores were applied as follows: 0, negative staining in all cells; 1+, weakly positive or focally positive staining in <10% of cells; 2+, moderately positive-staining in 10%-50% of cells; and score 3+, strongly positive-staining, involving 50% or more of the cells.

### Immunohistochemical Examinations

All patients were underwent a core needle biopsy before NAC, and had received a curative operation of a mastectomy or a conservative surgery with axillary lymph node dissection after NAC in Osaka City University. Tissues from each patient were fixed in buffered formalin and embedded in paraffin. Serial tissue sections with 4 µm were stained with hematoxylin-eosin and used for immunohistochemical staning. Expressions of CD133, estrogen receptor (ER), progesterone receptor (PgR), and HER2 were assessed by immunohistochemistry. After the paraffin sections were deparaffinized, they were heated for 20 min at 105°C by autoclave in Target Retrieval Solution (Dako, Carpinteria, CA). After blocking with 10% goat serum, the slides were incubated with each primary monoclonal antibody against, ER (clone 1D5, dilution 1∶80; Dako, Cambridge, UK), PgR (clone PgR636, dilution 1∶100; Dako), HER2 (Hercep Test, Dako), CD133 (clone NCH-38, dilution 1∶200; Dako), and ALDH1 (dilution 1∶100; BD Bioscience, San Jose, CA) overnight at 4°C. Peroxidase was introduced using a streptavidin conjugate and then peroxidase reactivity was visualized using a DAB solution, followed by counterstaining with haematoxylin.

**Table 2:** Correlations between CD133 or ALDH1 expression and clinicopathological parameters in CNB of 102 primary breast cancers.

		CD133			ALDH1	
Parameter	Posivive	Negative		Posivive	Negative	
	(n = 47)	(n = 55)	*p*-value	(n = 16)	(n = 86)	*p*-value
Age						
≥55	19	31		9	41	
<55	28	24	0.108	7	45	0.529
Menopose						
positive	32	39		12	59	
negative	15	16	0.757	4	27	0.771
Intrinsic subtype						
luminal A	20	26		3	43	
luminal B	4	4		2	6	
HER2	6	11	0.587	5	12	0.103
basal like	17	14		6	25	
Tumol size						
≥4 cm	8	10		4	14	
<4 cm	39	45	0.878	12	72	0.475
Lymph node status						
positive	20	13		5	28	
negative	27	42	0.042	11	58	0.918
Lymph-vascular invasion						
positive	21	7		2	27	
negative	26	48	0.001	14	59	0.145
Nuclear grade						
Grade 1&2	42	53		15	80	
Grade 3	5	2	0.244	1	6	0.916
Pathologiccal complete response						
pCR	9	21		5	25	
Non-pCR	38	34	0.035	11	61	0.860
Pathological response						
responder (Grade 2&3)	18	38		7	49	
non-responder (Grade 1)	29	17	0.002	9	37	0.329
Clinical response						
responder (cCR+cPR)	33	47		15	65	
non-responder (cNC+cPD)	14	8	0.062	1	21	0.183
Distant recurrence (metastasis)						
positive	21	7		6	22	
negative	26	48	<0.001	10	64	0.327

### Immunohistochemical Assessment

Immunohistochemical scoring was performed in a blind manner. The cut-off for ER positivity and PgR positivity was ≥1% positive tumor cells with nuclear staining. HER2 was graded according to the accepted grading scheme as 0, 1+, 2+, 3+. the following criteria were used for scoring: 0, no reactivity or membranous reactivity in less than 10% of cells; 1+, faint/barely perceptible membranous reactivity in 10% of cells or higher or reactivity in only part of the cell membrane; 2+, weak to moderate complete or basolateral membranous reactivity in 10% of tumor cells or higher; 3+, strong complete or basolateral membranous reactivity in 10% of tumor cells or higher. HER-2 was considered to be positive if immunostaining was 3+ or if a 2+ result showed gene amplication by fluorescent in situ hybridization (FISH). In FISH analyses, each copy of the *HER2* gene and its centromere 17 (*CEP17*) reference were counted. The interpretation followed the criteria of the ASCO/CAP guidelines for HER2 IHC interpretation for breast cancer [39]: positive if the *HER2*/CEP17 ratio was higher than 2.2. CD133 and ALDH1 antibody stained intensely the membrane and cytoplasm of cancer cells. Scores were applied as follows: score 0, negative staining in all cells; score 1+, weekly positive or focally positive staining in <10% of the cells; score 2+, moderately positive staining covering 10% to 50% of the cells; and score 3+, stringly positive staining, including >50% of the cells. CD133 and ALDH1 expression was considered positive when scores were ≥2.

### Statistical Analysis

Statistical analysis was performed using SPSS 13.0 statistical software (SPSS Inc, Chicago, USA). The association between the expression of CD133 or ALDH1 and clinicopathological parameters was analyzed with the chi-square test. The Kaplan-Meier method was used to estimate the values of DFS. DFS was compared using a Log-rank test. Events for the calculation of DFS included all local, regional, or distant recurrence. The Cox regression model was used for multivariate analysis of prognostic factors. In all of the tests, a *p* value less than 0.05 was considered to be statistically significant.

## Results

### Clinical and Pathological Response of Primary Breast Cancers to NAC

The cCR rate was 18% (18/102), cPR was 61% (62/102), cNC was 20% (20/102), and cPD was 2% (2/102). Clinical responders (cCR + cPR) included 78% (80/102) of the patients. Of the tumors investigated, 12% (12/102) were grade 1a, 33% (34/102) were grade 1b, 20% (20/102) were grade 2a, 16% (16/102) were grade 2b, and 20% (20/102) were grade 3. Patients were was classified according to the grade of tumor into pathologic responders, who had grade 2 and 3 tumors and equaled 55% of all patients, and non-responders, who had grade 1 tumors and equaled 45%. Overall, the pCR rate was 29% (30/102) **(** **Table 1** **)**. DFS in pathologic non-responders was significantly poorer (p = 0.01) than that in pathologic responders **(** **Fig. 1** **)**.

### Association between Clinicopathological Parameters and CD133 or ALDH1 Expression in Before NAC

Immunohistochemical patterns of CD133 or ALDH1 expression were analyzed in core needle biopsy (CNB) specimens from 102 patients. Forty-seven patients had CD133-positive primary breast tumors (46%) **(** **Fig. 2A** **)**, while 55 (54%) had CD133-negative tumors before NAC. Sixteen patients (16%) had ALDH1-positive primary breast tumors, while 86 patients (84%) had ALDH1-negative tumors before NAC **(** **Fig. 2B** **)**. **Table 2** shows the characteristics of patients included in the study. CD133 expression in CNB specimens significantly correlated with lymph node metastasis (43%, p = 0.042) and lymphatic invasion (45%, p<0.001). In contrast, no significant association was found between ALDH1 expression and clinicopathological factors. The pCR rate of CD133-positive tumors (19%, 9/47) was significantly lower (p = 0.035) than that of CD133-negative tumors (38%, 21/55). The pathological non-responder (grade 1) tumors were more frequently (p = 0.002) CD133 positive (69%, 38/55) than the pathological responder (grade 2 and 3) tumors (38%, 18/47). CD133 positivity was associated with the clinical response (cCR + cPR) (p = 0.062). Recurrence was observed in 28 patients and was more frequently found in patients with CD133-positive tumors (21/47, 45%) than those with CD133-negative tumors (7/55, 13%). There was no significant association between CD133 expression and other clinicopathological factors.

### Association between pCR and CD133 or ALDH1 Expression in CNB Specimens

Univariate and multivariate analyses of pCR of tumors before NAC are shown in **Table 3**. Univariate analysis revealed that CD133, ER, and PgR expression was significantly associated with pCR. However, multivariate analysis revealed that only CD133 expression was significantly associated with pCR.

**Table 3:** Univariate and multivariate analysis with pathological complete response in 102 breast cancers.

Parameter	Univarite analysis			Multivariate analysis		
	Odds ratio	95%CI	*p* value	Odds ratio	95%CI	*p* value
CD133 expression in CNB						
positive vs negative	0.38	0.16–0.95	0.038	0.15	0.29–0.80	0.027
ALDH1 expression in CNB						
positive vs negative	1.11	0.35–3.52	0.861			
ER						
positive vs negative	0.24	0.10–0.61	0.002	0.41	0.13–1.33	0.138
PgR						
positive vs negative	0.22	0.08–0.61	0.004	0.28	0.07–1.09	0.067
HER2						
positive vs negative	1.75	0.68–4.48	0.24			

### Association between Survival and CD133 or ALDH1 Expression

**Figure 3:**
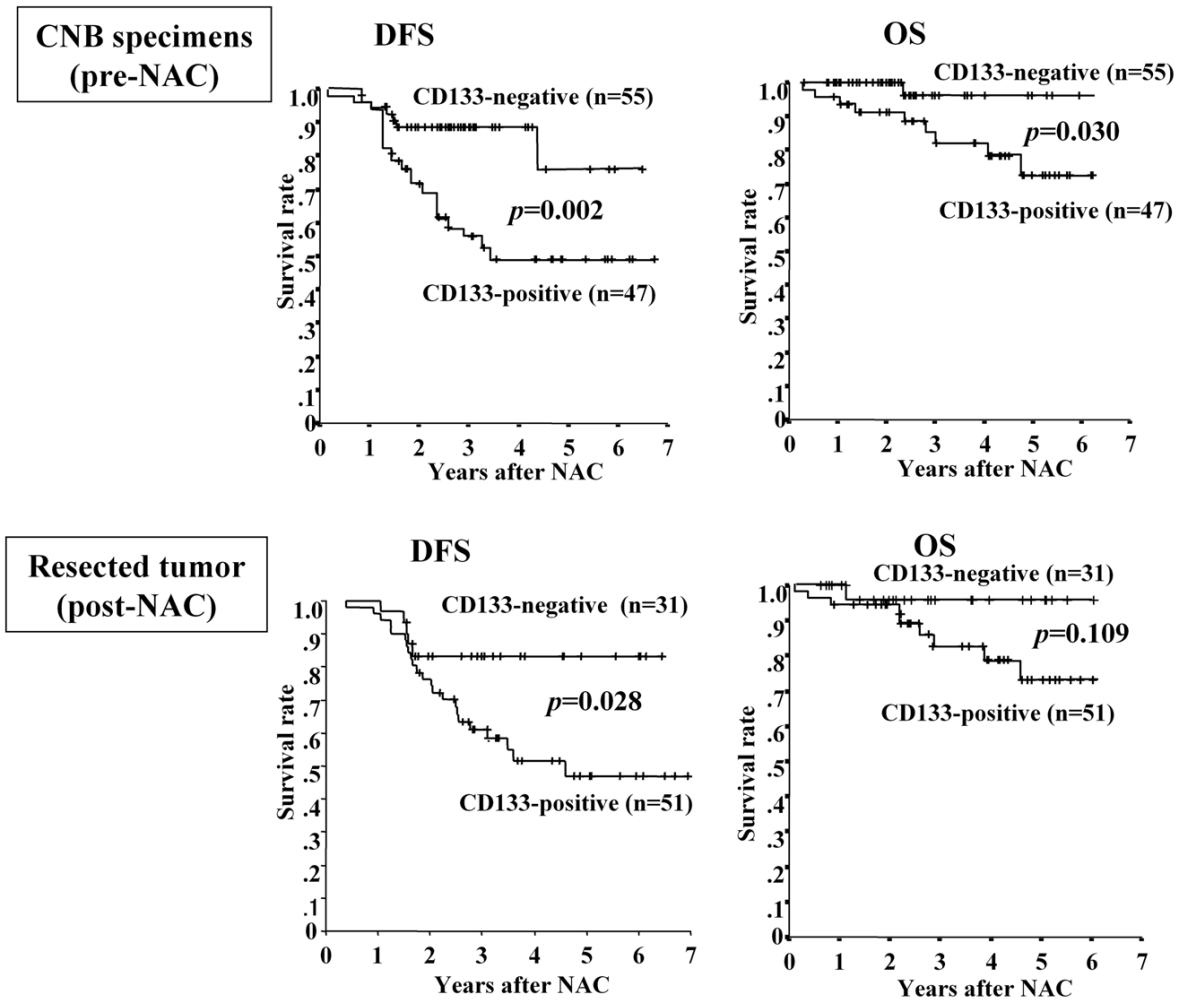
Association between CD133 expression and survival. In CNB specimens before NAC, DFS and OS in patients with CD133-positive tumors were significantly shorter than those in patients with CD133-negative tumors. In resected tumors after NAC**,** DFS for patients with CD133-positive tumors were significantly shorter than for patients with CD133-negative tumors, while OS was not significantly different. Since 20 of the 102 breast tumors were of pathological grade 3 before NAC, CD133 expression was examined in only 82 tumors after NAC.

DFS and OS in patients with CD133-positive tumors were significantly shorter (p = 0.002 and p = 0.030, respectively) than those in patients with CD133-negative tumors before NAC. In contrast, ALDH1 expression did not correlate with response to DFS and OS. Since 20 of the 102 breast tumors were of pathological grade 3 before NAC, CD133 expression was examined in only 82 tumors after NAC. DFS in patients with CD133-positive tumors was significantly shorter (p = 0.028) than that for patients with CD133-negative tumors after NAC. Univariate and multivariate regression analyses indicated that CD133 expression before NAC was an independent prognostic factor for DFS (**Table 4**). Although OS in patients with CD133-positive tumors was shorter than that in patients with CD133-negative tumors, this difference was not significantly different (p = 0.109, **Figure 3**).

**Table 4:** Univariate and multivariate analysis with respect to disease free survival in primary breast cancers.

Parameter	Univarite analysis			Multivariate analysis		
	Odds ratio	95%CI	*p* value	Odds ratio	95%CI	*p* value
CNB-CD133						
positive vs negative	3.63	1.54–8.55	0.003	2.56	1.01–6.48	0.046
CNB-ALDH1						
positive vs negative	1.53	0.62–3.78	0.357			
Tumor size						
≥4 cm vs <4 cm	2.40	1.09–5.30	0.031	2.15	0.92–5.01	0.076
Lymph node status						
N1–3 vs N0	3.08	1.46–6.52	0.003	1.17	0.47–2.90	0.736
Lymph-vascular invasion						
positive vs negative	4.23	2.00–8.96	<0.001	1.95	0.78–4.89	0.157
ER						
positive vs negative	0.94	0.44–2.00	0.872			
PgR						
positive vs negative	0.99	0.47–2.09	0.981			
HER2						
positive vs negative	1.32	0.58–3.01	0.51			
pCR						
positive vs negative	0.18	0.44–0.78	0.021	0.35	0.73–1.64	0.181

### Association between CD133 Expression Before and After NAC

**Figure 4:**
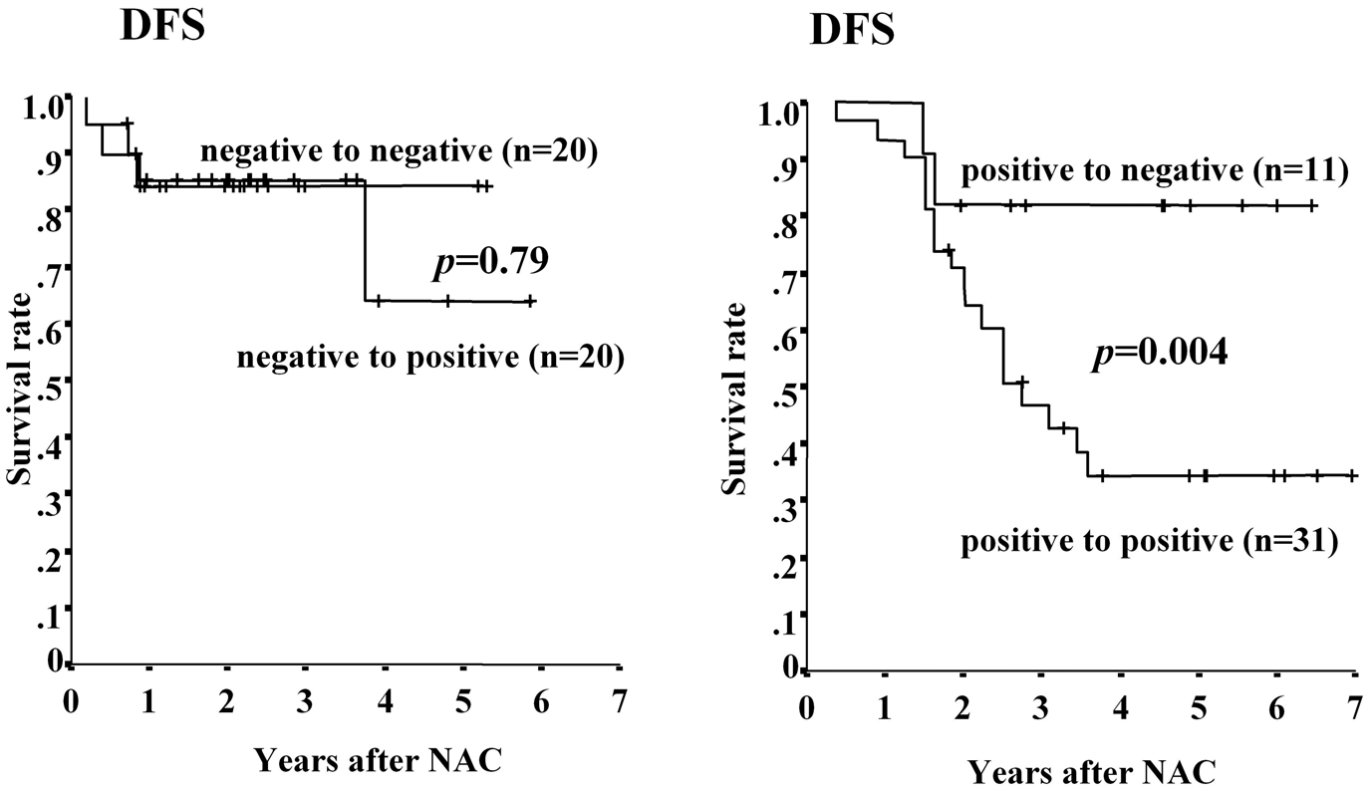
Association between CD133 expression and survival before and after NAC. DFS in patients CD133-positive tumors after NAC was significantly shorter than that for patients with CD133-neagtive tumors, while OS was not significant.

The association of CD133 expression between CD133 expression before and after NAC is shown in **Table 5**. The number of patients with CD133-positive tumors was higher after NAC (51/82; 62%) than that before NAC (47/102; 46%). Changes in CD133 expression were compared before and after NAC in the 82 patients who did not achieve pCR. Of these 82 patients, 42 patients had CD133-positive tumors and 40 had CD133-negative tumors before NAC. Of the 40 patients with CD133-negative tumors before NAC, 20 (50%) remained CD133 negative, whereas 20 (50%) changed to CD133 positive after NAC. Significantly more patients who were CD133-negative before and after NAC (18/20) were pathological responders of grade 2 (p<0.001). On the other hand, most patients who were CD133-positive before NAC showed the same status after NAC (31/42; 74%). Patients with CD133-negative tumors (7/11) were significantly more frequently pathological responders (p = 0.019) than patients with CD133-positive tumors. In contrast, tumor recurrence was more frequent in patients with CD133-positive (19/31) tumors before and after NAC **(** **Figure 4** **)**.

**Table 5:** CD133 Status between pre-NAC CNB specimens and post-NAC resected tumors.

	CNB-CD133-negative (n = 40)			CNB-CD133-positive (n = 42)		
Parameter	Ope-CD133			Ope-CD133		
	negative	positive		negative	positive	
	(n = 20)	(n = 20)	*p*-value	(n = 11)	(n = 31)	*p*-value
Pathological response						
responder (grade 2)	18	5		7	6	
non-responder (grade 0&1)	2	15	<0.001	4	25	0.019
Recurrence						
negative	17	16		9	12	
positive	3	4	0.677	2	19	0.032

## Discussion

In this study, NAC included FEC followed by PTX, which is a standard treatment regimen for patients with breast cancer [4], [5]. Despite the various definitions of pathological response in neoadjuvant trials, no significant differences in survival among the various classification systems, including cTMN, Fisher’s, Chevailler’s, and JBCS, has been reported. This study used the histological response criteria of WHO and JBCS. In this study, the pCR rate was 29% (30/102) and the cCR rate was 78% (80/102). These response rates were very similar to those previously reported [6], [9], [10]. NAC has numerous advantages, including the use of pathological response data as a surrogate marker for long-term clinical outcome [11], [12]. Forty-seven (46%) of patients had CD133-positive primary breast tumors before NAC, which was similar to findings from a previous study [40]. Before NAC, CD133 expression significantly associated with lymph node status and lymph-vascular invasion, as reported previously [23], [40], [41]. These findings may suggest that the function of CD133 is associated with lymph node metastasis.

The cCR rate of CD133-positive tumors was significantly lower than that of CD133-negative tumors. Multivariate analysis revealed that CD133 expression before NAC was an independent predictive factor for pCR. These results may indicate that CD133-positive tumors play a significant role in resistance to chemotherapy.

Recent progress in CSC research has led to a better understanding of the mechanism of resistance to chemotherapy and development of more effective chemotherapeutic regimens and new antitumor agents [42]. An association between CSCs and drug resistance in breast cancer cell lines has been shown *in vitro* [43]–[45]. In breast cancer, CD44^+^CD24^−/low^ [46], [47], aldehyde dehydrogenase (ALDH1) [48], [49], and CD133 [23]–[25] have been considered as markers of CSCs. However, recent studies have shown that CD44^+^CD24^−/low^ tumor cell numbers are not associated with pCR rates after NAC [50], [51]. In our study, ALDH1 expression did not correlate with pCR rates and response to DFS and OS. As previous report, ALDH1 expression in tumor cells did not correlate with response to neoadjuvant therapy, DFS, or OS after NAC [18]. CD133 may indicate CSC properties in a more restricted manner than other CSC markers such as CD44/CD24 or ALDH1 [52]. Although CD133 has been considered as a CSC marker in breast cancer [23]–[25], there has been no report regarding CD133 expression in breast cancer treated with NAC until date. In the present study, we found that CD133 expression before NAC may be a useful marker for predicting the effectiveness of NAC in breast cancer. To the best of our knowledge, this is the first study to report the potential clinical benefits of evaluating CD133 expression.

Correlative studies of tumor samples before and after treatment may provide further information on markers that could predict response or resistance to NAC; however, until date, few reports have examined pathological responses in tumors before and after chemotherapy [50]. Changes in CD133 expression before and after NAC were studied in 82 patients who did not achieve pathological grade 3. CD133 expression after NAC (62%) was higher than that before NAC (46%); thus, enrichment for CD133-positive cells was observed in post-NAC tumor specimens than in pre-NAC specimens. Most patients with CD133-positive tumors before NAC remained positive after NAC. These findings suggest that NAC was effective in reducing CD133-negative cells, and resulted in an increase in CD133-positive CSCs.

Distant recurrence was observed in 28 of 102 patients, and was significantly (p<0.001) frequent in patients with CD133-positive tumors (21/47, 45%). However, 7 of 28 patients who developed distant recurrence were CD133-negative. CD133 might not detect all of CSCs. Another CSC marker besides CD133 might be necessary to predict a distant recurrence of breast cancer patients after curative operation. In contrast, 26 of 47 patients with CD133-positive tumors did not develop distant recurrence within 7 years after operation. However, the 26 patients might have a possibility of recurrence in the future, because some breast cancer recurrent after the long disease-free interval. It might be necessary to continue the follow-up of distant recurrence for the 26 patients with CD133-positive tumors.

CD133 expression in after NAC was not correlated with prognosis, while before NAC it appeared to be associated with poor prognosis. Thus, CD133 expression after NAC may not be clinically informative for patients treated with NAC.

ER- or PR-negative patients were associated with higher pCR rates after NAC than in ER- or PR-positive patients, as previously reported [5], [17], while multivariate analysis showed that neither of these factors were independent surrogate markers for pCR. The response to NAC could be predicted more accurately by adding CD133 expression to that of ER and PR.

In conclusion, CD133 is a useful surrogate maker for predicting chemosensitivity and recurrence to FEC followed by PTX in breast cancer.
